# RAS signaling and remodeling of the immune microenvironment in pancreatic ductal adenocarcinoma: implications of emerging RAS-targeted therapy

**DOI:** 10.3389/fcell.2026.1948028

**Published:** 2026-08-25

**Authors:** Di Meng, Cheng Zhang

**Affiliations:** 1 Department of Traditional Chinese Medicine, The First Hospital of China Medical University, Shenyang, Liaoning, China; 2 Department of Pancreatic and Biliary Surgery, The First Hospital of China Medical University, Shenyang, Liaoning, China

**Keywords:** cancer-associated fibroblasts, immunosuppression, KRAS, pancreatic ductal adenocarcinoma, ras signaling, tumor immune microenvironment

## Abstract

Pancreatic ductal adenocarcinoma (PDAC) remains one of the most lethal malignancies. Its poor prognosis is closely related to early invasion, extensive stromal deposition, and a strongly immunosuppressive tumor microenvironment. Mutant KRAS is a major driver of PDAC initiation and progression, but its effects are not limited to cancer-cell proliferation. Increasing evidence indicates that oncogenic RAS signaling also alters the surrounding microenvironment by affecting fibroblast activation, extracellular matrix production, antigen presentation, myeloid-cell infiltration, and inflammatory cytokine signaling. These changes can restrict antitumor immune responses and may contribute to treatment resistance. As direct RAS-targeted agents move into clinical development, their effects on tumor immunity have become increasingly relevant. By integrating RAS-driven stromal and immune remodeling with emerging allele-specific and multi-selective RAS therapies, this Mini Review bridges mechanistic and clinical perspectives that are often discussed separately. This framework highlights the rationale for combining RAS inhibitors with immunotherapy and other microenvironment-targeted treatments.

## 1 Introduction

Pancreatic ductal adenocarcinoma (PDAC) is the most common type of pancreatic cancer and continues to carry a poor prognosis. Most patients are diagnosed too late for surgery, and recurrence remains frequent even after resection (Stoffel et al., 2023; Tonini and Zanni, 2025). Although FOLFIRINOX and gemcitabine plus nab-paclitaxel have improved treatment outcomes, the overall 5-year survival rate is still only about 13% (Kane et al., 2026). Immune checkpoint inhibitors (ICIs) have changed the treatment of melanoma, non-small cell lung cancer, and several other tumors (Shiravand et al., 2022), yet single-agent activity in PDAC is minimal. Major barriers include dense desmoplastic stroma, low tumor mutational burden, and extensive infiltration by immunosuppressive myeloid cells and regulatory lymphocytes. Microsatellite instability, a marker associated with ICI sensitivity, occurs in only about 1% of PDAC (Farhangnia et al., 2024). These features restrict immune-cell entry, antigen recognition, and effector function. Immunotherapy in PDAC will therefore probably require rational combinations with chemotherapy, radiotherapy, targeted agents, or other immune-modulating strategies. The choice of combination should reflect the dominant barrier in each tumor rather than apply one approach to all patients. Evidence from bladder and prostate cancer further indicates that the tumor microenvironment is a dynamic determinant of immunotherapy response and therapeutic resistance, shaped by regional immune architecture, immunometabolic reprogramming, and molecular heterogeneity (Fan et al., 2024; Wang et al., 2025d; Wang et al., 2025e).

KRAS is the dominant driver in PDAC and is mutated in more than 90% of cases. Most alterations involve codon 12: G12D accounts for about 40%, followed by G12V, G12R, G12C, and less common variants at Q61 or other sites (Singhal et al., 2024). Direct targeting was long considered impractical because RAS lacks a conventional binding pocket, binds GTP/GDP with high affinity, and activates partly redundant pathways. The success of covalent KRAS G12C inhibitors changed this view and accelerated development of KRAS G12D inhibitors, multi-selective RAS(ON) inhibitors, degraders, and neoantigen-directed immunotherapies (Huang et al., 2021).

Clinical data presented at the 2026 American Society of Clinical Oncology Annual Meeting showed a survival benefit with the multi-selective RAS inhibitor daraxonrasib (RMC-6236) in advanced pancreatic cancer (O’Reilly et al., 2026). RAS inhibition may also act beyond tumor cell-autonomous growth. Oncogenic KRAS influences fibroblasts, extracellular matrix, inflammatory mediators, antigen presentation, and myeloid-cell recruitment. Using CRISPR-mediated KRAS inactivation, Ischenko et al. (2021) found that KRAS-deficient PDAC cells could form tumors in immunodeficient mice but were efficiently cleared in immunocompetent syngeneic hosts, where they induced a strong antitumor response. This result links oncogenic KRAS to immune escape rather than proliferation alone. It also supports testing RAS inhibitors as tools to alter the tumor microenvironment and improve immunotherapy response.

## 2 Oncogenic RAS signaling in PDAC

RAS proteins are small GTPases that cycle between an active GTP-bound state and an inactive GDP-bound state (Simanshu et al., 2017). Growth-factor binding, for example, EGF binding to EGFR, induces receptor dimerization and phosphorylation. This activates guanine nucleotide exchange factors, which release GDP from RAS and permit GTP binding. Active RAS then engages several downstream pathways controlling proliferation, survival, metabolism, and inflammation. The signal is normally terminated by GTPase-activating proteins, which accelerate the intrinsic hydrolysis of GTP to GDP and return RAS to its inactive state (Keeton et al., 2017). In normal cells, RAS activation is transient and controlled by GDP–GTP exchange and GTP hydrolysis. Among the RAS isoforms, KRAS is the most frequently altered in PDAC. Mutations at codons 12, 13, or 61 reduce GTP hydrolysis and keep KRAS in an active state (Yang et al., 2023). Activated KRAS signals mainly through the RAF–MEK–ERK, PI3K–AKT–mTOR, and Ral–TBK1/NF-κB pathways, promoting proliferation, metabolic adaptation, survival, and inflammation (Singh and Smith, 2020; Mozzarelli et al., 2024; Wang X. et al., 2025). Crosstalk among these pathways also contributes to resistance when a single downstream target is inhibited.

## 3 RAS-driven immune microenvironment remodeling

### 3.1 Cancer-associated fibroblasts and desmoplastic stroma

Dense desmoplasia is a defining feature of PDAC. Cancer-associated fibroblasts (CAFs) and extracellular matrix form a physical and biochemical barrier around tumor cells. Oncogenic RAS activates Hedgehog signaling, and tumor-derived Sonic hedgehog promotes a fibroblast-rich stroma (Ji et al., 2007; Rhim et al., 2014). Hedgehog inhibition reduced stromal content, improved perfusion, and enhanced chemotherapy delivery in preclinical models (Olive et al., 2009; Zhao et al., 2018). KRAS-driven epithelial signaling may therefore maintain matrix deposition and restrict the access of both drugs and immune cells to the tumor core.

CAFs are heterogeneous rather than a single population. Öhlund et al. (2017) described alpha-SMA-high myofibroblastic CAFs (myCAFs) near tumor epithelium and more distant inflammatory CAFs (iCAFs) that secrete IL-6 and other cytokines. Their different locations reflect local signals from cancer cells. Tumor-derived IL-1 promotes the iCAF state through LIF and JAK/STAT signaling, whereas TGF-β suppresses IL-1R1 and favors myCAF differentiation (Biffi et al., 2019). Liu et al. (2024) found that, in KRAS-driven mouse models of PDAC, CAFs can in turn maintain MYC expression in PDAC cells and weaken the effect of RAS pathway inhibitors. Kuo et al. (2025) established a mouse model of pancreatic cancer with KRAS mutation and Arid1a loss and found that these alterations jointly increased immune cell infiltration and reduced stromal activation. This observation argues against viewing every KRAS-mutant tumor as uniformly fibrotic and immune excluded. The epithelial genotype, local cytokine gradients, and treatment exposure together determine the balance between myCAF and iCAF programs. These differences are clinically relevant because depletion of all fibroblasts may remove both tumor-promoting and tumor-restraining functions.

Direct KRAS inhibition can reverse part of this stromal program. Across 16 KRAS G12D-driven PDAC models, MRTX1133 reprogrammed CAFs and increased the activity of immune checkpoint blockade (Mahadevan et al., 2023). In immunocompetent implantable and autochthonous models, the drug induced major regression within 14 days, with complete or near-complete responses in some tumors (Kemp et al., 2023). These responses involved apoptosis and proliferative arrest, but also changes in fibroblasts, extracellular matrix, and macrophages. RAS signaling therefore links the intrinsic malignant state of PDAC cells to the surrounding stromal and immune compartments. Overall, RAS-directed therapy may be most effective when it reprograms, rather than indiscriminately depletes, the heterogeneous CAF compartment to improve drug delivery and antitumor immunity.

### 3.2 Antigen presentation and T-cell recognition

Effective antitumor T-cell immunity depends on efficient processing of tumor antigens by tumor cells or antigen-presenting cells and their presentation through MHC molecules. The MHC-I antigen presentation pathway mainly involves proteasomal degradation of endogenous proteins, transport of antigenic peptides by TAP1/TAP2, loading of peptides onto MHC-I heavy chains in the endoplasmic reticulum, and transport of peptide-MHC-I complexes to the cell surface for recognition by CD8^+^ T cells (Wang et al., 2024). Studies have shown that RAS and downstream MAPK signaling can suppress MHC-I-related antigen presentation on the tumor cell surface, whereas blockade of RAS/MAPK signaling can partially restore MHC-I expression and antigen presentation capacity (El-Jawhari et al., 2014).

Persistent KRAS activation in PDAC may reduce tumor cell immune visibility, making it difficult for CD8^+^ T cells to recognize and kill tumor cells effectively. McAndrews et al. (2025) reported that inhibition of the RAS pathway in immunocompetent mouse models increased intratumoral CD8^+^ effector T-cell infiltration, reduced myeloid cell infiltration, and rendered tumors responsive to immune checkpoint blockade. Xu et al. (2025) constructed lipid nanoparticles that co-delivered KRAS G12D mutant mRNA neoantigen and the STING agonist cGAMP. This system activated type I interferon signaling, upregulated MHC-I, CD80, and CD86 expression in hepatic antigen-presenting cells, promoted IFN-γ-producing CD8^+^ cytotoxic T cells, significantly suppressed the growth of liver metastases, and prolonged animal survival. These findings suggest that mRNA delivery targeting KRAS-mutant neoantigens combined with STING activation can remodel the immune microenvironment of PDAC metastases by enhancing antigen presentation and T-cell effector function, although the evidence remains preclinical. In 16 preclinical models of KRAS G12D-driven PDAC, Mahadevan et al. (2023) found that MRTX1133 inhibited early PDAC growth and induced regression of established PanIN lesions and advanced PDAC. Its antitumor effect was not only related to KRAS G12D inhibition but was also accompanied by increased CD8^+^ effector T-cell infiltration, reduced myeloid infiltration, and CAF reprogramming. Mechanistically, MRTX1133 induced FAS expression in tumor cells and enhanced FAS/FASL-related tumor cell killing mediated by CD8^+^ T cells. Orlen et al. (2025) found that the RAS(ON) multi-selective inhibitors daraxonrasib and RMC-7977 induced rapid and marked tumor regression in immunocompetent PDAC mouse models, accompanied by reduced myeloid cells and increased T cells and macrophages. The depth and durability of tumor regression depended on T cells and conventional dendritic cells, indicating that the antitumor effect after RAS(ON) inhibition is closely related to restoration of antigen presentation and T-cell immunity. Hashimoto et al. (2019) found that KRAS upregulates ARF6 and AMAP1 through eIF4A/eIF4E-dependent translation. The ARF6-AMAP1 pathway further participates in PD-L1 recycling and promotes immune evasion by tumor cells. Overall, RAS inhibition may restore tumor immune visibility by enhancing antigen presentation and CD8^+^ T-cell function, providing a mechanistic rationale for combination with immune checkpoint blockade or antigen-directed immunotherapies.

### 3.3 Recruitment and expansion of suppressive cells

RAS signaling also changes the immune-cell composition of PDAC through cytokines, chemokines, and extracellular vesicles. These signals recruit myeloid-derived suppressor cells (MDSCs), promote tumor-associated macrophage polarization, and sustain neutrophil-mediated T-cell exclusion.

In KRAS-mutant PDAC, early-infiltrating macrophages release TIMP-1, which signals through CD63 on cancer cells and supports a subpopulation with reduced epithelial features, lymphangiogenesis, and immune escape (Wang C.-A. et al., 2025). Single-cell and spatial analyses indicate that this macrophage-cancer-cell circuit emerges early and may contribute to later metastatic behavior. KRAS-TP53 co-mutant tumor cells can also produce CXCL1, recruiting CXCR2^+^ neutrophil-like MDSCs that release TNF and exclude T cells from tumor nests (Bianchi et al., 2023). The effect of KRAS, however, depends on cooperating alterations. Rnf43 loss in a KRAS G12D model reduced macrophages, increased T and B cell infiltration, and raised immune-checkpoint expression (Hosein et al., 2022). This immune-rich state may create different therapeutic vulnerabilities from those of conventional stroma-dense PDAC. A further mechanism involves extracellular KRAS G12D. Dai et al. (2020) found that pancreatic cancer cells can release KRAS G12D protein through exosomes under oxidative stress. Extracellular KRAS G12D is taken up by macrophages through an AGER-dependent mechanism and induces M2-like pro-tumor macrophage polarization through STAT3-dependent fatty acid oxidation, thereby promoting pancreatic cancer progression. Blocking KRAS G12D release or uptake weakens macrophage-mediated pro-tumor effects, and KRAS G12D levels in macrophages are associated with poor prognosis in patients with pancreatic cancer. Therefore, future therapeutic strategies may need to combine RAS inhibition with myeloid cell reprogramming, chemokine-axis blockade, and immune checkpoint therapy to improve the sensitivity of PDAC to immunotherapy. Thus, RAS inhibition alone may be insufficient to reverse myeloid-dominant immunosuppression, supporting combinations with myeloid reprogramming or chemokine-axis blockade.

### 3.4 Activation of pro-tumor inflammatory cytokine networks

Oncogenic RAS promotes a persistent inflammatory state in PDAC through several interconnected pathways. In KRAS G12D models, Ling et al. (2012) found that KRAS G12D induces IL-1α expression through AP-1 and thereby activates IKK2/β-NF-κB signaling. NF-κB further induces IL-1α and p62 expression, forming an IL-1α/p62 positive feedback loop that maintains sustained NF-κB activation. The study also showed that high IL-1α expression is associated with KRAS mutation, increased NF-κB activity, and poor prognosis in human PDAC, suggesting that KRAS does not drive tumor cell proliferation in isolation but maintains a pro-tumor microenvironment through inflammatory feed-forward mechanisms. In addition, inhibition of NF-κB signaling can significantly reduce KRAS-driven tumorigenesis (Feng et al., 2024).

**FIGURE 1:**
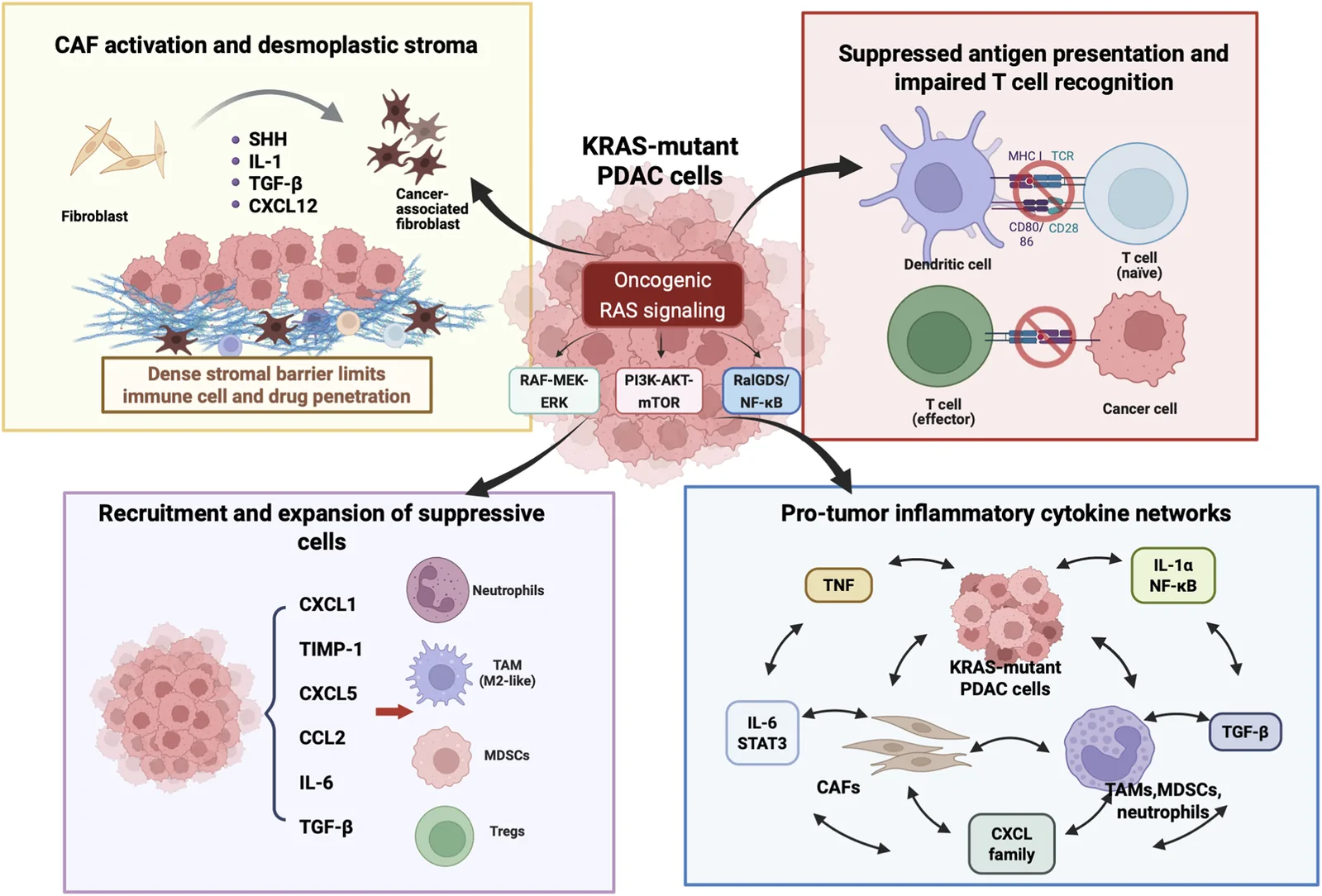
Oncogenic RAS signaling remodels the immune microenvironment in PDAC. In KRAS-mutant PDAC cells, activation of the RAF–MEK–ERK, PI3K–AKT–mTOR, and RalGDS–NF-κB pathways promotes four interconnected processes: CAF activation and desmoplastic stroma; impaired antigen presentation and T-cell recognition; recruitment and expansion of neutrophils, M2-like tumor-associated macrophages, myeloid-derived suppressor cells, and regulatory T cells; and pro-tumor inflammatory cytokine networks involving tumor cells, CAFs, and myeloid cells.

IL-6/STAT3 is another important pathway. IL-6 trans-signaling promotes progression from pancreatic intraepithelial neoplasia to PDAC and supports tumor-cell growth, invasion, and survival (Lesina et al., 2011). It also weakens dendritic-cell function and favors MDSC and macrophage accumulation (van Duijneveldt et al., 2020). IL-6 blockade can reduce several KRAS-driven effects in experimental models (Song et al., 2024). In time-resolved single-cell analysis of a KRAS G12D model, epithelial and stromal inflammatory programs were accompanied by dysfunctional myeloid and CD4^+^ T-cell states in premalignant lesions (Schlesinger et al., 2020). Early intervention may therefore differ biologically from treatment of established PDAC. RAS can also alter immune-costimulatory pathways. CD137 normally supports activated T and natural killer cells (Lee et al., 2025), but KRAS upregulates CD137 on PDAC cells through MAPK and IL-1α/NF-κB signaling (Glorieux and Huang, 2019). The function of tumor-cell CD137 is not equivalent to its costimulatory role on lymphocytes. Inflammation is not uniformly tumor promoting, however. Nanoparticle delivery of STING/TLR4 agonists with senescence-inducing therapy activated type I interferon, improved antigen presentation, and produced T-cell-dependent regression in PDAC models (Chibaya et al., 2024). Overall, RAS-driven inflammation is context dependent but generally sustains immune exclusion and treatment resistance, supporting selective blockade of tumor-promoting cytokine circuits while preserving antitumor inflammation. Together, these four mechanisms show that oncogenic RAS creates interconnected stromal and immune barriers, whereas RAS blockade may partially reverse them (Figure 1). This mechanistic framework provides the rationale for the therapeutic agents and combination strategies discussed in the following section.

## 4 Breakthroughs in RAS-Targeted therapy

### 4.1 KRAS G12C inhibitors

KRAS G12C was the first KRAS subtype successfully targeted in the clinic. Sotorasib and adagrasib covalently bind the inactive GDP-bound protein and block downstream signaling. In previously treated KRAS G12C-mutant pancreatic cancer, the CodeBreaK 100 study reported a 21% objective response rate, median progression-free survival of 4.0 months, and median overall survival of 6.9 months with sotorasib (Strickler et al., 2023). Later-line divarasib produced a 27% response rate and median progression-free survival of 7.1 months (Krebs et al., 2025). Early results with HRS-7058 included responses in three of four patients with PDAC and disease control in all four, although this sample is too small for firm conclusions (Huang et al., 2025). A phase II study of HRS-7058 after prior systemic therapy is ongoing (NCT07589569). These data establish proof of principle, but KRAS G12C occurs in only a small fraction of PDAC.

### 4.2 KRAS G12D inhibitors

KRAS G12D is much more common in PDAC and could extend targeted treatment to a larger group. The selective non-covalent inhibitor MRTX1133 showed strong preclinical activity, but its clinical program ended before phase II and mature efficacy data are unavailable (Wei et al., 2024; Xu et al., 2026). HRS-4642, a selective long-acting KRAS G12D inhibitor, showed preliminary activity alone and with gemcitabine plus nab-paclitaxel in phase Ib/II testing; no treatment discontinuations due to adverse events were reported (Wang L. et al., 2025). GFH375 inhibits both GDP-bound and GTP-bound KRAS G12D. At ESMO 2025, once-daily monotherapy showed early activity in previously treated advanced PDAC (NCT06500676) (Zhou et al., 2025). These findings are encouraging, but most evidence comes from small open-label cohorts or conference abstracts. The ongoing phase III study (NCT07262567) will provide a more reliable assessment of benefit and toxicity.

### 4.3 Pan-RAS inhibitors

ADT-007 is a pan-RAS inhibitor that binds nucleotide-free RAS and prevented nucleotide loading and signal activation in preclinical studies. It suppressed KRAS-mutant PDAC cells and inhibited tumor growth in mouse models (Foote et al., 2025). Because the parent compound has low water solubility and limited exposure, the oral prodrug ADT-1004 was developed. ADT-1004 is converted to ADT-007 *in vivo* and has shown activity across several KRAS variants, together with changes in the immune microenvironment (Bandi et al., 2025). These results remain preclinical, and its therapeutic window in patients is unknown. Daraxonrasib (RMC-6236) is an oral, non-covalent, multi-selective RAS(ON) inhibitor. It forms a complex with cyclophilin A and active GTP-bound RAS, preventing interaction with downstream effectors. Its broader coverage includes common PDAC variants such as G12D, G12V, and G12R and may avoid the narrow eligibility of allele-specific drugs. In the phase I/II RMC-6236–001 study (NCT05379985), second-line daraxonrasib 300 mg produced a 35% objective response rate, median progression-free survival of 8.5 months, and median overall survival of 13.1 months in RAS G12-mutant PDAC (Wolpin et al., 2026). The randomized phase III RASolute 302 trial (NCT06625320) subsequently compared daraxonrasib with investigator-selected chemotherapy after first-line treatment. Median overall survival was 13.2 months with daraxonrasib and 6.7 months with standard chemotherapy (O’Reilly et al., 2026). These results support multi-selective RAS(ON) inhibition as a strategy for a much larger PDAC population than KRAS G12C-directed therapy. Questions remain about resistance mechanisms, activity after other RAS inhibitors, and the most effective combination partners. Daraxonrasib is still investigational; expanded access, breakthrough therapy designation, and orphan drug designation do not constitute regulatory approval or establish it as standard care.

## 5 Discussion

RAS-targeted therapy is beginning to change the treatment landscape of PDAC. Daraxonrasib (RMC-6236), a multi-selective RAS(ON) inhibitor, has shown clinical activity in previously treated RAS-mutant metastatic disease and improved survival compared with standard chemotherapy in a randomized phase III trial. These findings support broader molecular stratification in PDAC. Treatment choice should consider the KRAS variant, co-mutations, and prior therapy rather than histology alone. Chemotherapy remains the mainstay, but combining RAS inhibitors with gemcitabine plus nab-paclitaxel, FOLFIRINOX-based regimens, or radiotherapy may enhance efficacy by weakening survival signaling and modifying the tumor microenvironment (Rozengurt and Eibl, 2026).

RAS inhibitors may also remodel the immunosuppressive microenvironment of PDAC. By reducing suppressive cytokines and myeloid-cell recruitment, they may improve T-cell infiltration and increase sensitivity to immune checkpoint blockade. This provides a rationale for combining RAS inhibitors with PD-1/PD-L1 or CTLA-4 blockade, cancer vaccines, and T-cell–redirecting therapies. From the perspective of combination therapy, oncoMUC-driven immunosuppression may be an important adaptive mechanism that limits the efficacy of KRAS G12D inhibitors in residual tumor cells after RAS inhibition. Bhyravbhatla et al. (2026) found that MRTX1133 treatment upregulated oncoMuc4 and oncoMuc16 expression, whereas pharmacological targeting of oncoMUCs enhanced the antitumor effect of MRTX1133 and reduced the expression of immune checkpoints such as VISTA, TIM-3, and PD-L1. Broderick et al. (2025) proposed a combination strategy using a CDK4/6 inhibitor and a CD40 agonist. This combination induced durable tumor regression and established CD4^+^ T-cell-dependent tumor-immune equilibrium. However, when RAS inhibitors are combined with immunotherapy, overlapping immune-related adverse events and targeted-drug toxicities should be carefully monitored. Several limitations should be considered. Most evidence for RAS-mediated immune remodeling remains preclinical, while longitudinal immune profiling in patients is limited. PDAC heterogeneity, rapidly evolving clinical data, and the lack of validated biomarkers further restrict interpretation and patient selection, underscoring the need for larger prospective trials.

## 6 Conclusion

RAS signaling supports PDAC growth and contributes to immune suppression through stromal remodeling, impaired antigen presentation, myeloid-cell accumulation, and inflammatory signaling. RAS inhibition may weaken these barriers and improve responses to chemotherapy or immunotherapy. Future studies should clarify immune effects in patients, define predictive biomarkers, and optimize combination regimens with acceptable toxicity. However, because many immune-remodeling data remain preclinical and several clinical signals are preliminary, the efficacy, safety, and biomarker-defined applicability of these strategies require confirmation in larger prospective trials.
